# Supplementary material for: Clinical and Genetic Characteristics of Covid-19 Patients from UK Biobank
Source: medRxiv. 2020 May 5:2020.05.05.20075507. Preprint. [Version 1] doi: 10.1101/2020.05.05.20075507 (PMC7276016; doi:10.1101/2020.05.05.20075507)
Supplement: 1 [file 79272-2020.05.05.20075507-1.docx]

**Supplementary Appendix**

CONTENTS

| Supplementary Methods | Page 2 |
| --- | --- |
| Supplementary Table 1 | Page 3 |
| Supplementary Table 2 | Page 5 |
| Supplementary Table 3 | Page 6 |
| Supplementary Table 4 | Page 8 |
| Supplementary Table 5 | Page 9 |
| Supplementary Table 6 | Page 10 |
| Supplementary Table 7 | Page 11 |
| Supplementary Figure 1 | Page 12 |
| Supplementary Figure 2 | Page 13 |
| Supplementary Figure 3 | Page 14 |
| Supplementary Figure 4 | Page 15 |
| Supplementary Figure 5  Supplementary Figure 6 | Page 16  Page 17 |
| References | Page 18 |
|  |  |

**Supplementary Methods**

UK Biobank is a cohort of participants who were recruited from 2006 to 2010. Data on baseline characteristics were obtained during an in-person interview, and health outcome data were obtained prospectively. Written informed consent was obtained from all study participants.

UK Biobank is prospectively collecting Covid-19 results from March 16, 2020 onwards, including information on specimen date, specimen type, processing laboratory, inpatient status, and test results. Most UK testing performed during this period was completed in hospital. Samples were categorized as inpatient if they were retrieved from an emergency care provider, an inpatient location, or if the sample was related to healthcare associated infection.

Frequencies and percentages were calculated for each of three categories: all participants, participants with only negative tests, and participants with a minimum of one positive test. We then calculated risk ratios for categorical variables, and we used quasi-Poisson regression to estimate risk ratios for continuous variables. Risk ratios for age, body-mass index, and systolic blood pressure were calculated by determining the change in risk for each ten unit increase in the risk factor. Adjusted estimates were controlled for age, sex, body-mass index, systolic blood pressure, ethnicity, and Townsend deprivation index.

Covid-19 host genetic analyses were investigated through a series of genome wide association studies. ABO blood types were inferred from the variants rs8176719 and rs8176746, as previously described.^1^ We analyzed the inferred values through logistic regression adjusted for age, sex, body-mass index, ethnicity and Townsend deprivation index. Next, genome wide association analyses implemented logistic regression for each genetic variant against Covid-19 status, corrected for age, sex, and the first ten principal components of genetics. Lastly, we constructed polygenic risk scores, calculated as the sum of the product between the number of effect alleles and externally estimated variant effect size. Additional details of the methods are provided in the caption of each respective figure.

*Study Outcome*

The main study outcome was any positive test for a single individual. We also used simple descriptive statistics to explore the frequency of testing results and types of tests used amongst all tests performed on the cohort.

In order to better understand risk factors for inpatient and outpatient Covid-19, we also conducted a nested case-control study within the population of patients that tested positive for Covid-19 on at least one occasion. In order to better understand risk factors for severe disease, we calculated odds ratios for inpatient care. Patients were categorized as inpatient if they had any Covid-19 testing sample marked as inpatient.

**Supplementary Table 1.** Risk Ratios for Baseline Characteristics for Participants with a Minimum of One Positive Covid-19 Test Relative to Participants with No Positive Test

| **Characteristic** | **Relative Risk**  **(95% CI)** | **P Value** | **Adjusted Relative Risk**^4^  **(95% CI)** | **P Value** |
| --- | --- | --- | --- | --- |
| **Baseline and demographic** |  |  |  |  |
| Age* | 1.16 (1.05 - 1.28) | 0.003 | 1.31 (1.18 - 1.45) | <0.001 |
| Sex – male vs. female | 1.59 (1.36 - 1.85) | <0.001 | 1.59 (1.35 - 1.87) | <0.001 |
| Body-mass index*^,1^ | 1.85 (1.62 - 2.10) | <0.001 | 1.77 (1.53 - 2.05) | <0.001 |
| Systolic blood pressure* ­ | 1.04 (1.00 - 1.08) | 0.04 | 0.98 (0.94 - 1.03) | 0.45 |
| Race – vs. white |  |  |  |  |
| Asian | 2.21 (1.56 - 3.14) | <0.001 | 2.03 (1.40 - 2.95) | <0.001 |
| Black | 4.35 (3.24 - 5.83) | <0.001 | 3.09 (2.25 - 4.25) | <0.001 |
| Townsend deprivation index | 1.13 (1.10 - 1.15) | <0.001 | 1.10 (1.08 - 1.13) | <0.001 |
| **Social habit** |  |  |  |  |
| Smoking – vs. never |  |  |  |  |
| Previous | 1.52 (1.29 - 1.79) | <0.001 | 1.41 (1.19 - 1.68) | <0.001 |
| Current | 1.29 (1.00 - 1.67) | 0.05 | 1.15 (0.88 - 1.50) | 0.32 |
| Alcohol use – vs. never |  |  |  |  |
| Once or twice a week | 0.57 (0.44 - 0.75) | <0.001 | 0.79 (0.59 - 1.05) | 0.11 |
| Three or four times a week | 0.52 (0.40 - 0.69) | <0.001 | 0.78 (0.58 - 1.06) | 0.12 |
| Daily or almost daily | 0.53 (0.40 - 0.70) | <0.001 | 0.73 (0.54 - 1.00) | 0.05 |
| **Comorbidity** |  |  |  |  |
| Cancer | 1.13 (0.86 - 1.47) | 0.39 | 1.16 (0.88 - 1.54) | 0.30 |
| Diabetes | 2.12 (1.65 - 2.74) | <0.001 | 1.19 (0.90 - 1.58) | 0.26 |
| Chronic obstructive pulmonary disease^2^ | 1.97 (1.36 - 2.89) | 0.001 | 1.54 (1.02 - 2.31) | 0.04 |
| Asthma | 1.38 (1.11 - 1.70) | 0.004 | 1.34 (1.07 - 1.67) | 0.009 |
| Ischemic heart disease^3^ | 2.19 (1.67 - 2.86) | <0.001 | 1.56 (1.18 - 2.07) | 0.002 |
| Hypothyroidism | 0.93 (0.64 - 1.33) | 0.79 | 0.92 (0.62 - 1.37) | 0.68 |
| Hypercholesterolemia | 1.46 (1.19 - 1.79) | <0.001 | 1.09 (0.88 - 1.36) | 0.43 |
| Allergic rhinitis | 1.11 (0.81 - 1.52) | 0.50 | 1.27 (0.93 - 1.75) | 0.14 |
| Depression | 1.55 (1.18 - 2.04) | 0.003 | 1.51 (1.13 - 2.02) | 0.005 |
| **Serology** |  |  |  |  |
| White blood cell count | 1.03 (1.02 - 1.04) | <0.001 | 1.03 (1.02 - 1.05) | <0.001 |
| Red blood cell count | 1.43 (1.19 - 1.72) | <0.001 | 0.96 (0.77 - 1.19) | 0.69 |
| Hemoglobin concentration | 1.06 (1.00 - 1.13) | 0.07 | 0.96 (0.89 - 1.04) | 0.33 |
| Mean corpuscular volume | 0.99 (0.97 - 1.00) | 0.08 | 1.00 (0.99 - 1.02) | 0.68 |
| Mean corpuscular hemoglobin concentration | 0.87 (0.81 - 0.95) | 0.001 | 0.92 (0.84 - 1.00) | 0.04 |
| Platelet count | 1.00 (1.00 - 1.00) | 0.13 | 1.00 (1.00 - 1.00) | 0.73 |
| Lymphocyte count | 1.03 (1.02 - 1.04) | <0.001 | 1.04 (1.02 - 1.06) | <0.001 |
| Monocyte count | 1.20 (1.08 - 1.26) | <0.001 | 1.21 (1.10 - 1.33) | <0.001 |
| Neutrophil count | 1.10 (1.04 - 1.15) | <0.001 | 1.06 (1.01 - 1.12) | 0.03 |

*Risk ratios are presented per 10 unit increase in risk factor.

^1^Body-mass index is the weight in kilograms divided by the square of the height in meters.

^2^Chronic obstructive pulmonary disease was defined as a diagnosis of emphysema and/or bronchitis.

^3^Ischemic heart disease was categorized as history of myocardial infarction or angina.

^4^Adjusted estimates were controlled for age, sex, body-mass index, systolic blood pressure, race, and Townsend deprivation score.

**Supplementary Table 2.** Risk Ratios for Medication Use for Participants with a Minimum of One Positive Covid-19 Test Relative to Participants with No Positive Test

| **Medication Class** | **Relative Risk (95% CI)** | **P Value** | **Adjusted Relative Risk (95% CI)**^8^ | **P Value** |
| --- | --- | --- | --- | --- |
| Non-steroidal anti-inflammatory drug^1^ | 1.17 (0.99 - 1.39) | 0.10 | 1.02 (0.86 - 1.22) | 0.79 |
| Angiotensin converting enzyme inhibitor^2^ | 1.76 (1.28 - 2.41) | 0.001 | 1.32 (0.95 - 1.84) | 0.10 |
| Angiotensin II receptor blocker^3^ | 1.84 (1.30 - 2.61) | 0.002 | 1.37 (0.94 - 1.98) | 0.10 |
| Dihydropyridine calcium channel blocker^4^ | 1.99 (1.54 - 2.57) | <0.001 | 1.28 (0.98 - 1.69) | 0.07 |
| Beta blocker^5^ | 1.68 (1.30 - 2.17) | <0.001 | 1.29 (0.98 - 1.69) | 0.07 |
| Thiazolidinedione^6^ | 1.74 (0.65 - 4.64) | 0.30 | 0.94 (0.35 - 2.57) | 0.91 |
| Sulfonylurea^7^ | 2.86 (1.77 - 4.62) | <0.001 | 1.49 (0.90 - 2.49) | 0.12 |
| **Other Common Therapies** |  |  |  |  |
| Acetaminophen | 1.31 (1.10 - 1.57) | 0.004 | 1.25 (1.03 - 1.51) | 0.02 |
| Levothyroxine | 1.00 (0.68 - 1.48) | 0.92 | 0.90 (0.59 - 1.39) | 0.65 |
| Metformin | 2.52 (1.85 - 3.43) | <0.001 | 1.24 (0.88 - 1.75) | 0.22 |
| Glucosamine | 0.73 (0.51 - 1.04) | 0.10 | 0.82 (0.56 - 1.18) | 0.28 |
| Cod liver oil capsule | 0.79 (0.55 - 1.15) | 0.24 | 0.90 (0.62 - 1.31) | 0.59 |

^1^Non-steroidal anti-inflammatory drugs included aspirin, ibuprofen, diclofenac, naproxen, indomethacin, celecoxib, and meloxicam.

^2^Angiotensin converting enzyme inhibitors included captopril, enalapril, lisinopril, fosinopril, ramipril, and quinapril.

^3^Angiotensin II receptor blockers included losartan, candesartan, eprosartan, irbesartan, olmesartan, telmisartan, and valsartan.

^4^Dihydropyridine calcium channel blockers included amlodipine, felodipine, isradipine, nicardipine, and nifedipine.

^5^Beta blockers included acebutolol, atenolol, bisoprolol, carvedilol, labetalol, metoprolol, nadolol, nebivolol, pindolol, and propranolol.

^6^Thiazolidinediones included rosiglitazone, troglitazone, and pioglitazone.

^7^Sulfonylureas included glipizide, glibenclamide, glibornuride, gliclazide, gliquidone, acetohexamide, tolbutamide, chlorpropamide, and tolazamide.

^8^Adjusted estimates were controlled for age, sex, body-mass index, systolic blood pressure, race, and Townsend deprivation score.

**Supplementary Table 3.** Characteristics of Covid-19 Positive Inpatients and Outpatients

| **Characteristic** | **Covid-19 Positive Outpatients**  **(N=95)** | **Covid-19 Positive Inpatients**  **(N=574)** |
| --- | --- | --- |
| **Baseline and demographic** |  |  |
| Mean age (years) | 56.8 (9.5) | 57.6 (8.5) |
| Male, no. (%) | 54 (56.8) | 324 (56.4) |
| Mean body-mass index, s.d.* | 28.7 (5.5) | 29.2 (5.5) |
| Mean systolic blood pressure, s.d. (mmHg) | 143.0 (23.6) | 141.1 (19.9) |
| Race, no. (%) |  |  |
| White | 74 (77.9) | 491 (86.0) |
| Asian | 5 (5.3) | 28 (4.9) |
| Black | 14 (14.7) | 34 (6.0) |
| Mean Townsend deprivation index, s.d. | 0 (3.8) | 0.1 (3.5) |
| **Social habit** |  |  |
| Smoking, no. (%) |  |  |
| Never | 40 (42.1) | 263 (46.1) |
| Previous | 41 (43.2) | 244 (42.7) |
| Current | 14 (14.7) | 58 (10.2) |
| Alcohol use, no. (%) |  |  |
| Never | 9 (9.5) | 75 (13.3) |
| Once or twice a week | 20 (21.1) | 137 (24.0) |
| Three or four times a week | 16 (16.8) | 112 (19.6) |
| Daily or almost daily | 15 (15.8) | 98 (17.2) |
| **Comorbidity** |  |  |
| Cancer, no. (%) | 6 (6.3) | 52 (9.1) |
| Diabetes, no. (%) | 10 (10.5) | 56 (9.8) |
| Chronic obstructive pulmonary disease, no. (%)^1^ | 4 (4.2) | 24 (4.2) |
| Asthma, no. (%) | 17 (17.9) | 85 (14.8) |
| Ischemic heart disease, no. (%)^2^ | 9 (9.5) | 49 (8.5) |
| Hypothyroidism, no. (%) | 3 (3.2) | 27 (4.7) |
| Hypercholesterolemia, no. (%) | 13 (13.7) | 98 (17.1) |
| Allergic rhinitis, no. (%) | 4 (4.2) | 38 (6.6) |
| Depression, no. (%) | 5 (5.3) | 51 (8.9) |
| **Serology** |  |  |
| Mean white blood cell count, s.d. | 7.0 (1.9) | 7.5 (5.6) |
| Mean red blood cell count, s.d. | 4.5 (0.5) | 4.6 (0.5) |
| Mean hemoglobin concentration, s.d. | 14.0 (1.8) | 14.3 (1.4) |
| Mean corpuscular volume, s.d. | 90.6 (7.1) | 90.8 (4.7) |
| Mean corpuscular hemoglobin concentration, s.d. | 34.2 (1.1) | 34.4 (1.0) |
| Mean platelet count, s.d. | 252.0 (73.6) | 249.1 (63.1) |
| Mean lymphocyte count, s.d. | 2.0 (0.7) | 2.3 (5.2) |
| Mean monocyte count, s.d. | 0.5 (0.2) | 0.5 (0.4) |
| Mean neutrophil count, s.d. | 4.3 (1.5) | 4.4 (1.6) |

*Body-mass index is the weight in kilograms divided by the square of the height in meters.

^1^Chronic obstructive pulmonary disease was defined as a diagnosis of emphysema and/or bronchitis.

^2^Ischemic heart disease was categorized as history of myocardial infarction or angina.

**Supplementary Table 4.** Frequency of Medication Use in Covid-19 Positive Inpatients and Outpatients

| **Medication Class** | **Covid-19 Positive Outpatients**  **(N=95)** | **Covid-19 Positive Inpatients**  **(N=574)** |
| --- | --- | --- |
| Non-steroidal anti-inflammatory drug, no. (%)^1^ | 33 (34.7) | 163 (28.4) |
| Angiotensin converting enzyme inhibitor, no. (%)^2^ | 9 (9.4) | 32 (5.6) |
| Angiotensin II receptor blocker, no. (%)^3^ | 7 (7.4) | 26 (4.5) |
| Dihydropyridine calcium channel blocker, no. (%)^4^ | 10 (10.5) | 55 (9.6) |
| Beta blocker, no. (%)^5^ | 8 (8.4) | 57 (9.9) |
| Thiazolidinedione, no. (%)^6^ | 0 (0) | 4 (0.7) |
| Sulfonylurea, no. (%)^7^ | 0 (0) | 17 (3.0) |
| **Other Common Therapies** |  |  |
| Acetaminophen, no. (%) | 16 (16.8) | 138 (24.0) |
| Levothyroxine, no. (%) | 6 (6.3) | 21 (3.7) |
| Metformin, no. (%) | 5 (5.3) | 38 (6.6) |
| Glucosamine, no. (%) | 3 (3.2) | 29 (5.1) |
| Cod liver oil capsule, no. (%) | 3 (3.2) | 27 (4.7) |

^1^Non-steroidal anti-inflammatory drugs included aspirin, ibuprofen, diclofenac, naproxen, indomethacin, celecoxib, and meloxicam.

^2^Angiotensin converting enzyme inhibitors included captopril, enalapril, lisinopril, fosinopril, ramipril, and quinapril.

^3^Angiotensin II receptor blockers included losartan, candesartan, eprosartan, irbesartan, olmesartan, telmisartan, and valsartan.

^4^Dihydropyridine calcium channel blockers included amlodipine, felodipine, isradipine, nicardipine, and nifedipine.

^5^Beta blockers included acebutolol, atenolol, bisoprolol, carvedilol, labetalol, metoprolol, nadolol, nebivolol, pindolol, and propranolol.

^6^Thiazolidinediones included rosiglitazone, troglitazone, and pioglitazone.

^7^Sulfonylureas included glipizide, glibenclamide, glibornuride, gliclazide, gliquidone, acetohexamide, tolbutamide, chlorpropamide, and tolazamide.

**Supplementary Table 5.** Odds Ratios for Baseline Characteristics for Inpatient Covid-19 Relative to Outpatient Disease

| **Characteristic** | **Odds Ratio (95% CI)** | **P Value** |
| --- | --- | --- |
| **Baseline and demographic** |  |  |
| Age* | 1.11 (0.87 - 1.43) | 0.39 |
| Sex – male vs. female | 0.98 (0.63 - 1.53) | 1.00 |
| Body-mass index*^,1^ | 1.20 (0.81 - 1.84) | 0.38 |
| Systolic blood pressure* ­ | 0.96 (0.86 - 1.07) | 0.41 |
| Race – vs. white |  |  |
| Asian | 0.84 (0.32 - 2.25) | 0.79 |
| Black | 0.37 (0.19 - 0.71) | 0.004 |
| Mean Townsend deprivation index | 1.00 (0.94 - 1.07) | 0.93 |
| **Social habit** |  |  |
| Smoking – vs. never |  |  |
| Previous | 0.91 (0.57 - 1.45) | 0.72 |
| Current | 0.63 (0.32 - 1.23) | 0.19 |
| Alcohol use – vs. never |  |  |
| Once or twice a week | 0.82 (0.36 - 1.90) | 0.84 |
| Three or four times a week | 0.84 (0.35 - 2.00) | 0.83 |
| Daily or almost daily | 0.78 (0.33 - 1.89) | 0.66 |
| **Comorbidity** |  |  |
| Cancer | 1.48 (0.62 - 3.56) | 0.44 |
| Diabetes | 0.93 (0.46 - 1.90) | 0.85 |
| Chronic obstructive pulmonary disease^2^ | 0.99 (0.34 - 2.93) | 1.00 |
| Asthma | 0.79 (0.45 - 1.41) | 0.44 |
| Ischemic heart disease^3^ | 0.89 (0.42 - 1.88) | 0.70 |
| Hypothyroidism | 1.51 (0.45 - 5.09) | 0.79 |
| Hypercholesterolemia | 0.70 (0.70 - 2.42) | 0.46 |
| Allergic rhinitis | 1.61 (0.56 - 4.63) | 0.50 |
| Depression | 1.76 (0.68 - 4.52) | 0.32 |

*Risk ratios are presented per 10 unit increase in risk factor.

^1^Body-mass index is the weight in kilograms divided by the square of the height in meters.

^2^Chronic obstructive pulmonary disease was defined as a diagnosis of emphysema and/or bronchitis.

^3^Ischemic heart disease was categorized as history of myocardial infarction or angina.

**Supplementary Table 6.** Odds Ratios for Medication Use for Inpatient Covid-19 Relative to Outpatient Disease

| **Medication Class** | **Odds Ratio (95% CI)** | **P Value** |
| --- | --- | --- |
| Non-steroidal anti-inflammatory drug^1^ | 0.75 (0.47 - 1.18) | 0.22 |
| Angiotensin converting enzyme inhibitor^2^ | 0.56 (0.26 - 1.22) | 0.16 |
| Angiotensin II receptor blocker^3^ | 0.60 (0.25 - 1.42) | 0.30 |
| Dihydropyridine calcium channel blocker^4^ | 0.91 (0.44 - 1.84) | 0.71 |
| Beta blocker^5^ | 1.20 (0.55 - 2.60) | 0.85 |
| Thiazolidinedione^6^ | – | ­– |
| Sulfonylurea^7^ | – | – |
| **Other Common Therapies** |  |  |
| Acetaminophen | 1.56 (0.88 - 2.76) | 0.15 |
| Levothyroxine | 0.56 (0.22 - 1.43) | 0.25 |
| Metformin | 1.28 (0.49 - 3.33) | 0.82 |
| Glucosamine | 1.63 (0.49 - 5.47) | 0.60 |
| Cod liver oil capsule | 1.51 (0.45 - 5.09) | 0.79 |

^1^Non-steroidal anti-inflammatory drugs included aspirin, ibuprofen, diclofenac, naproxen, indomethacin, celecoxib, and meloxicam.

^2^Angiotensin converting enzyme inhibitors included captopril, enalapril, lisinopril, fosinopril, ramipril, and quinapril.

^3^Angiotensin II receptor blockers included losartan, candesartan, eprosartan, irbesartan, olmesartan, telmisartan, and valsartan.

^4^Dihydropyridine calcium channel blockers included amlodipine, felodipine, isradipine, nicardipine, and nifedipine.

^5^Beta blockers included acebutolol, atenolol, bisoprolol, carvedilol, labetalol, metoprolol, nadolol, nebivolol, pindolol, and propranolol.

^6^Thiazolidinediones included rosiglitazone, troglitazone, and pioglitazone.

^7^Sulfonylureas included glipizide, glibenclamide, glibornuride, gliclazide, gliquidone, acetohexamide, tolbutamide, chlorpropamide, and tolazamide.

**Supplementary Table 7.** Odds Ratios for Blood Type for Participants with a Minimum of One Positive Covid-19 Test Relative to Participants with No Positive Test

| **Blood Type Compared to** **(%)*** | **Blood Type** | **Odds Ratio (95% CI)** | **P Value** |
| --- | --- | --- | --- |
| A (43.4) | B | 0.88 (0.66 - 1.14) | 0.31 |
|  | O | 0.77 (0.65 - 0.92) | 0.003 |
| B (9.6) | A | 1.15 (0.88 - 1.51) | 0.31 |
|  | O | 0.88 (0.67 - 1.17) | 0.40 |
| O (43.3) | A | 1.30 (1.09 - 1.54) | 0.003 |
|  | B | 1.13 (0.85 - 1.49) | 0.40 |

*The remaining 3.7% of the population with genotypes available were either an ambiguous blood type or had missing alleles that prevented a blood type inference.

**Supplementary Figure 1.** Genome Wide Association Study for the Nested Analysis of Inpatient Status for Participants with Any Positive Covid-19 Test, HLA and Specific Gene Boundaries


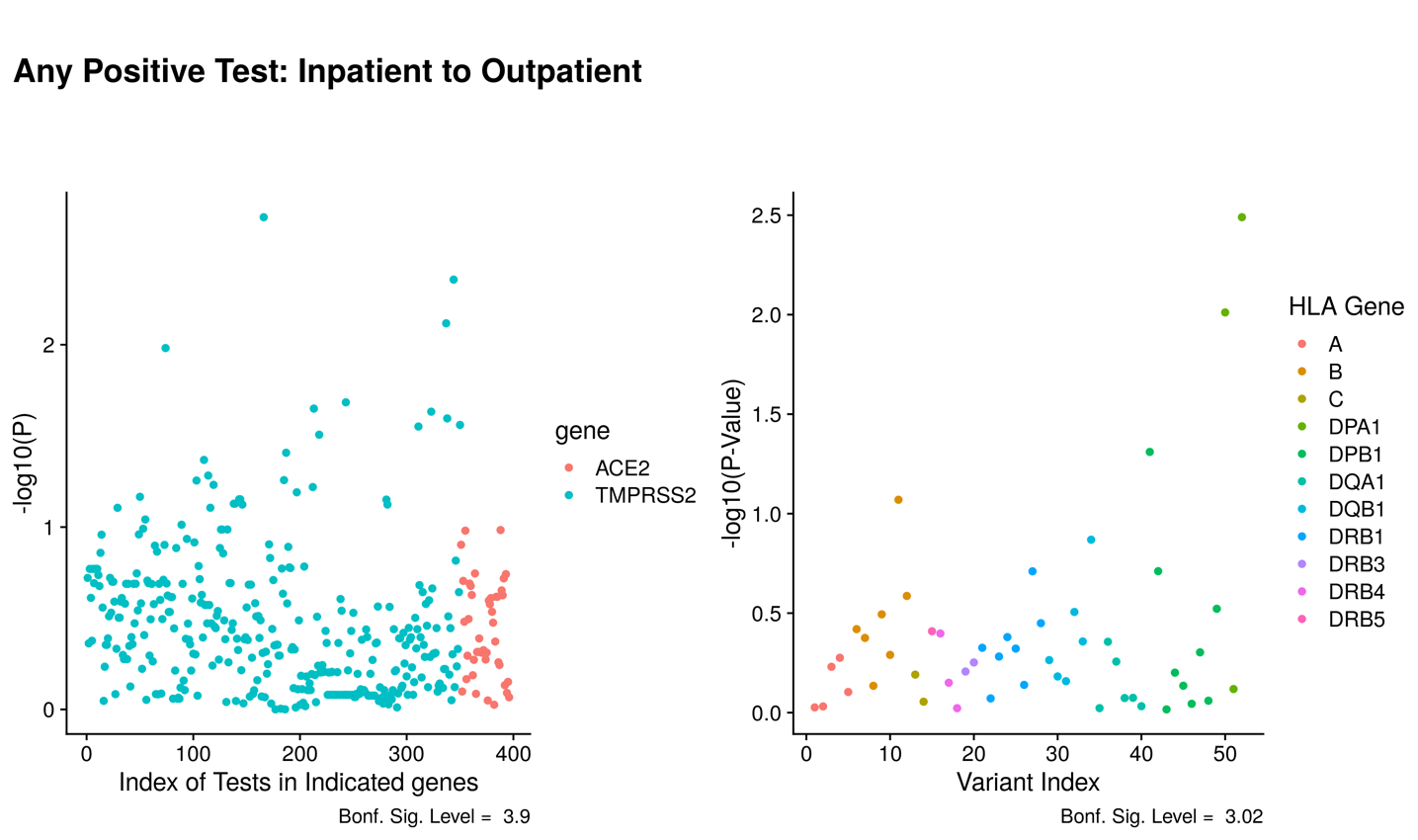


A

B

Shown are excerpts from the genome wide association study described in Supplementary Figure 4. Supplementary Figure 1A displays the tests which occurred within the boundaries of two genes of note: ACE2 and TMPRSS2. Supplementary Figure 1B shows tests of the Human Leukocyte Antigen (HLA Region) by conducting a similar series of logistic regression analyses between UK Biobank provided imputation values and the status of inpatient status within the positive testing population, corrected for age, sex, and the first 10 genetic principal components. Imputed variants with fewer than 10 unique values were excluded from analysis. The Bonferroni significance level, on the -log_10_ scale, is indicated within each figure.

**Supplementary Figure 2.** Genome Wide Association Study for Any Positive Covid-19 Test, HLA and Specific Gene Boundaries

A

B


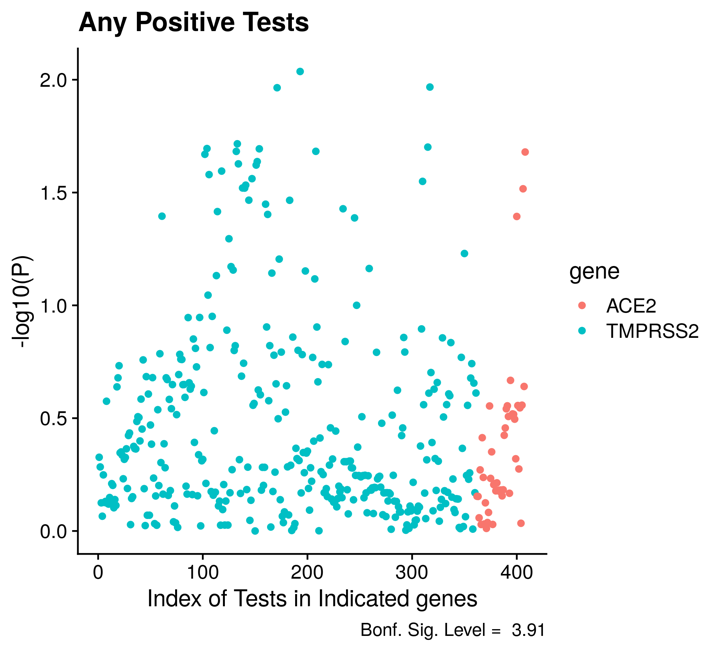

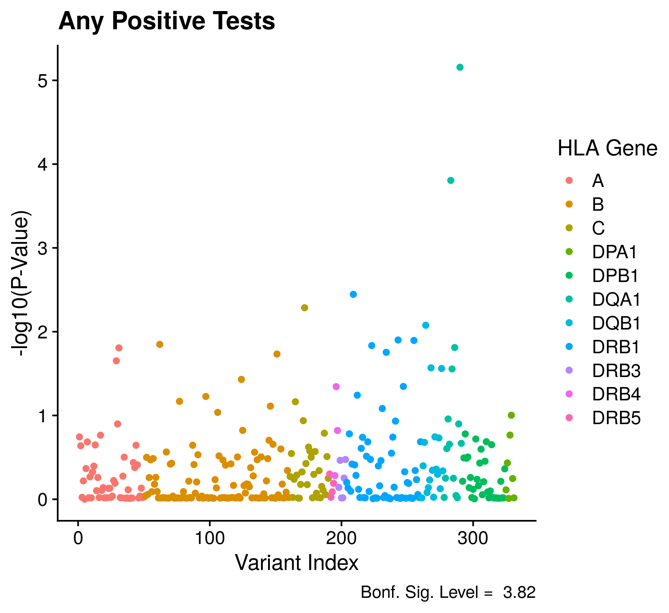


Shown are excerpts from the genome wide association study presented in Supplementary Figure 3. Supplementary Figure 2A displays the tests which occurred within the boundaries of two genes of note: ACE2 and TMPRSS2. Supplementary Figure 2B shows tests of the Human Leukocyte Antigen (HLA Region) through a similar series of logistic regression analyses between UK Biobank provided imputation values and positive Covid-19 testing results, corrected for age, sex, and the first 10 genetic principal components. Imputed variants with fewer than 10 unique values were excluded from analysis. The Bonferroni significance level, on the -log_10_ scale, is indicated within each figure.

**Supplementary Figure 3.** Genome Wide Association Study for Participants with Any Positive Covid-19 Test


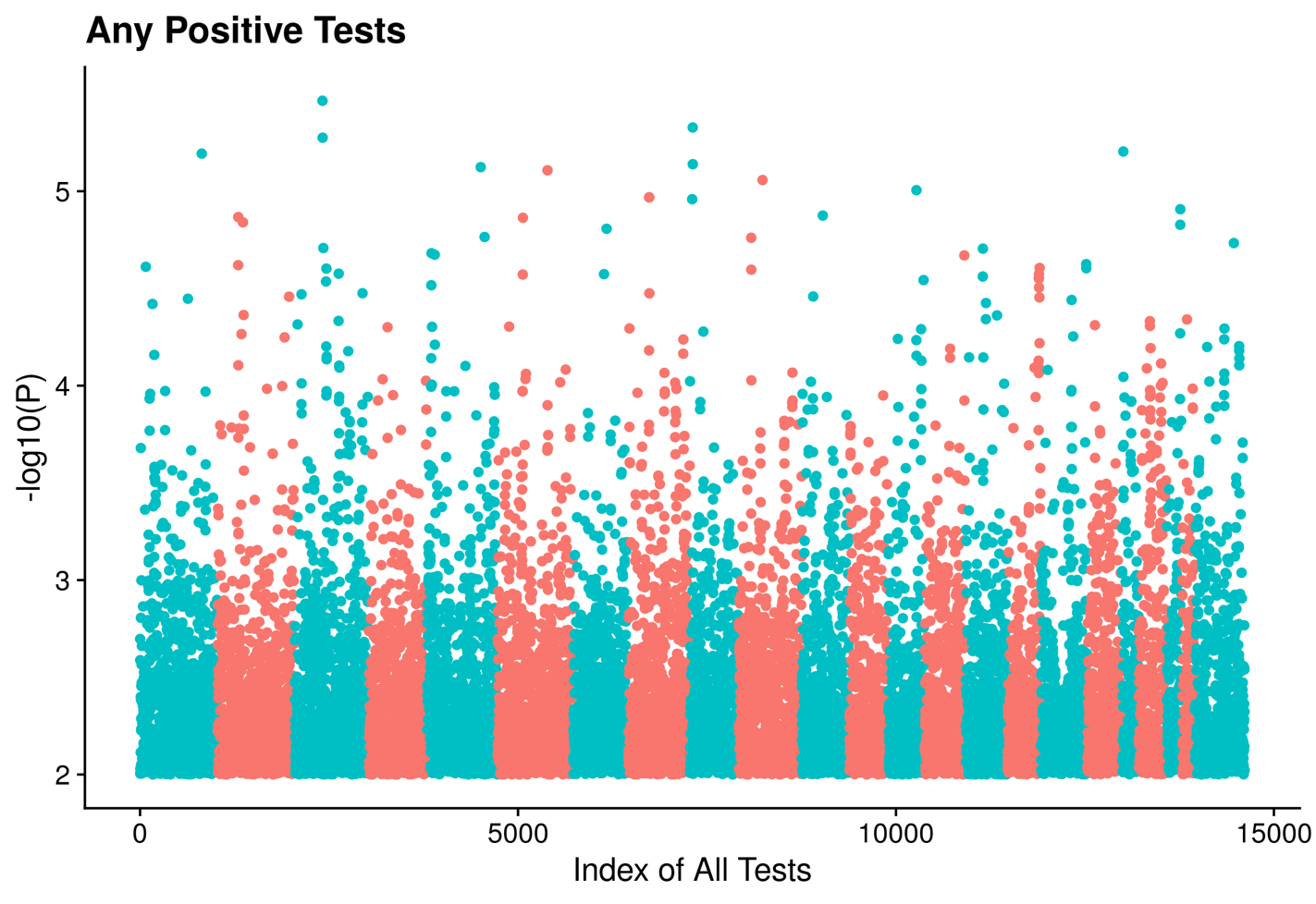


The genome wide association study was conducted on all UK Biobank individuals tested for Covid-19 with genotyping data. Specifically, there were 481,583 controls (no positive test) and 669 cases (at least one positive test). In total, 1,393,020 imputed variants, which all had minor allele frequency greater than 0.001 and were included in the HapMap Project, were tested through logistic regression corrected for age, sex, and the first 10 genetic principal components. The chromosomes are ordered numerically from 1 on the left to X on the right, with each progressive color change representing a different chromosome. The significance level employed was P = 5 x 10^-8^.

**Supplementary Figure 4.** Genome Wide Association Study for the Nested Analysis of Inpatient Status of Individuals with Any Positive Covid-19 Test


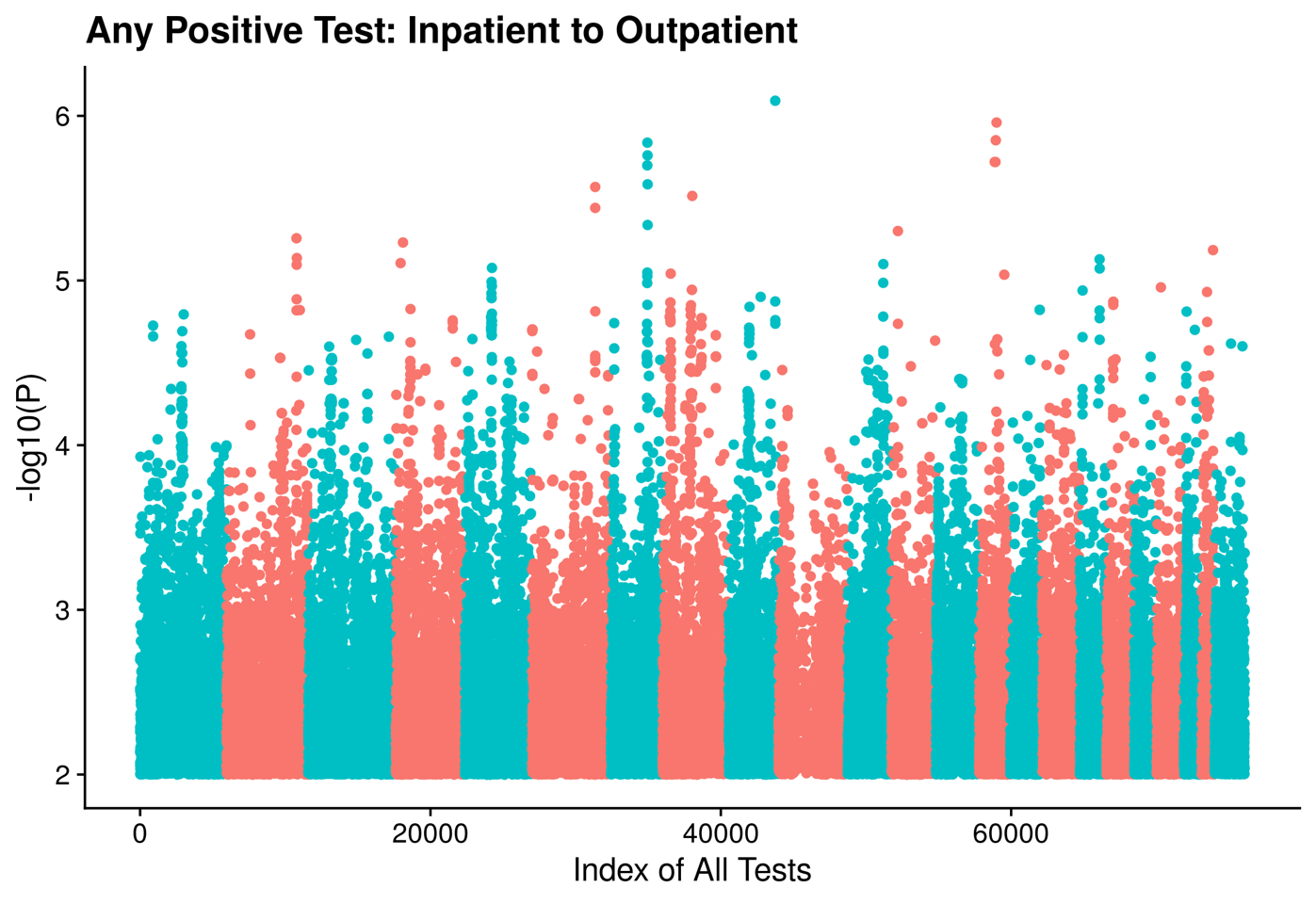


The genome wide association study was conducted on all UK Biobank individuals that tested positive for Covid-19 with genotyping data, comparing inpatient to outpatient status. There were 95 controls (outpatients) and 574 cases (inpatients). In total, 9,507,787 imputed variants, which all had minor allele frequency greater than 0.01 and passed basic quality control, were tested through logistic regression corrected for age, sex, and the first 10 genetic principal components. The chromosomes are ordered numerically from 1 on the left to X on the right, with each progressive color change representing a different chromosome. The significance level employed was P = 5 x 10^-8^.

**Supplementary Figure 5.** Stratification of the Cohort of Individuals Through Polygenic Risk Scores of Any Positive Covid-19 Test


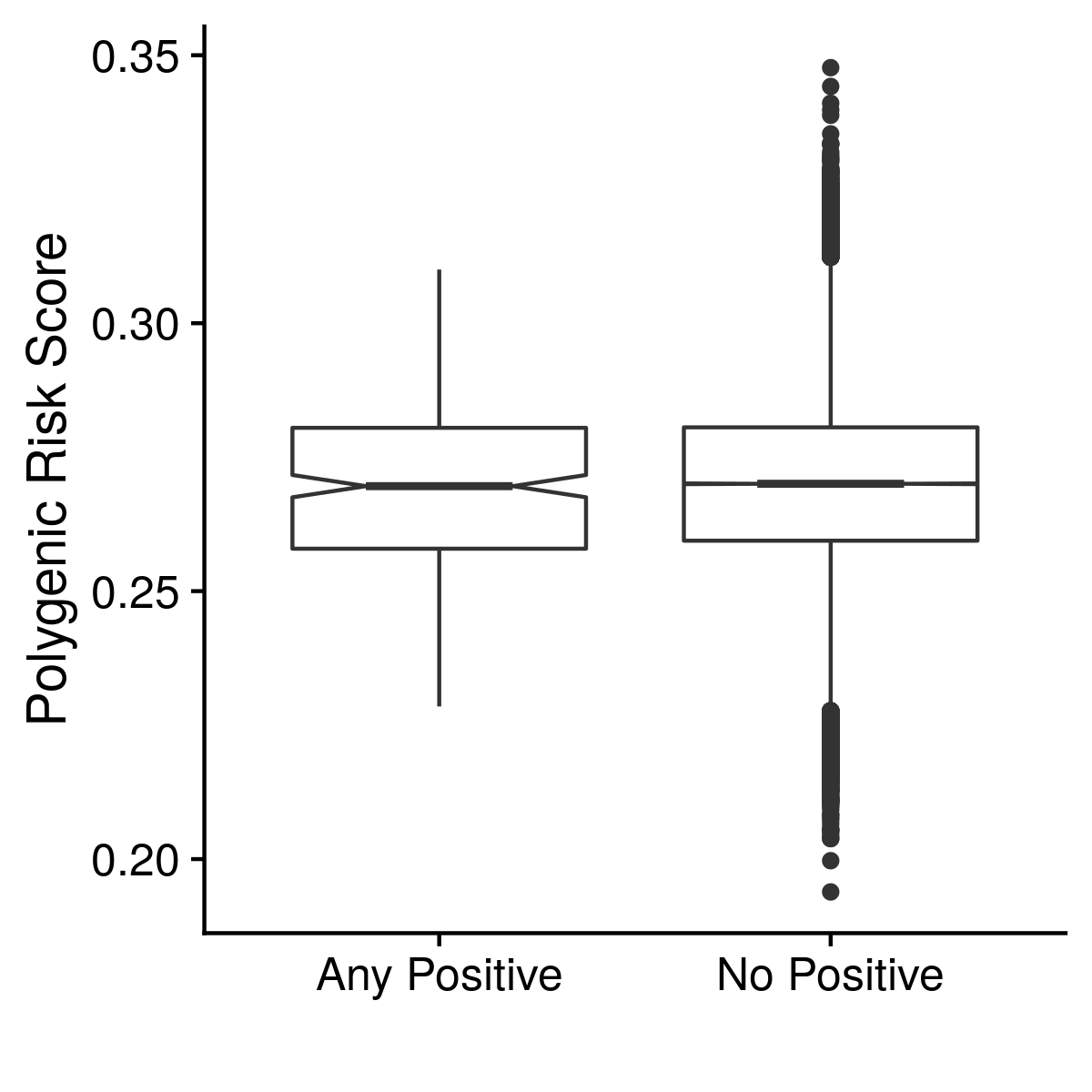


The entire population of individuals with genetic data were split into training (249,641 controls and 359 cases) and testing (231,942 controls and 310 cases) datasets. The training data was used to perform a genome wide association study as previously described. The result summary statistics were clumped with a p-value threshold of 0.01 and linkage disequilibrium R^2^ threshold of 0.5. Polygenic risk scores generated for the testing dataset and the status of any positive test result were compared. The difference between the polygenic risk scores of the any positive and no positive groups was determined to be insignificant (P = 0.92, T-test).

**Supplementary Figure 6.** Stratification of Individuals with Any Positive Result Through Polygenic Risk Scores of Inpatient Status


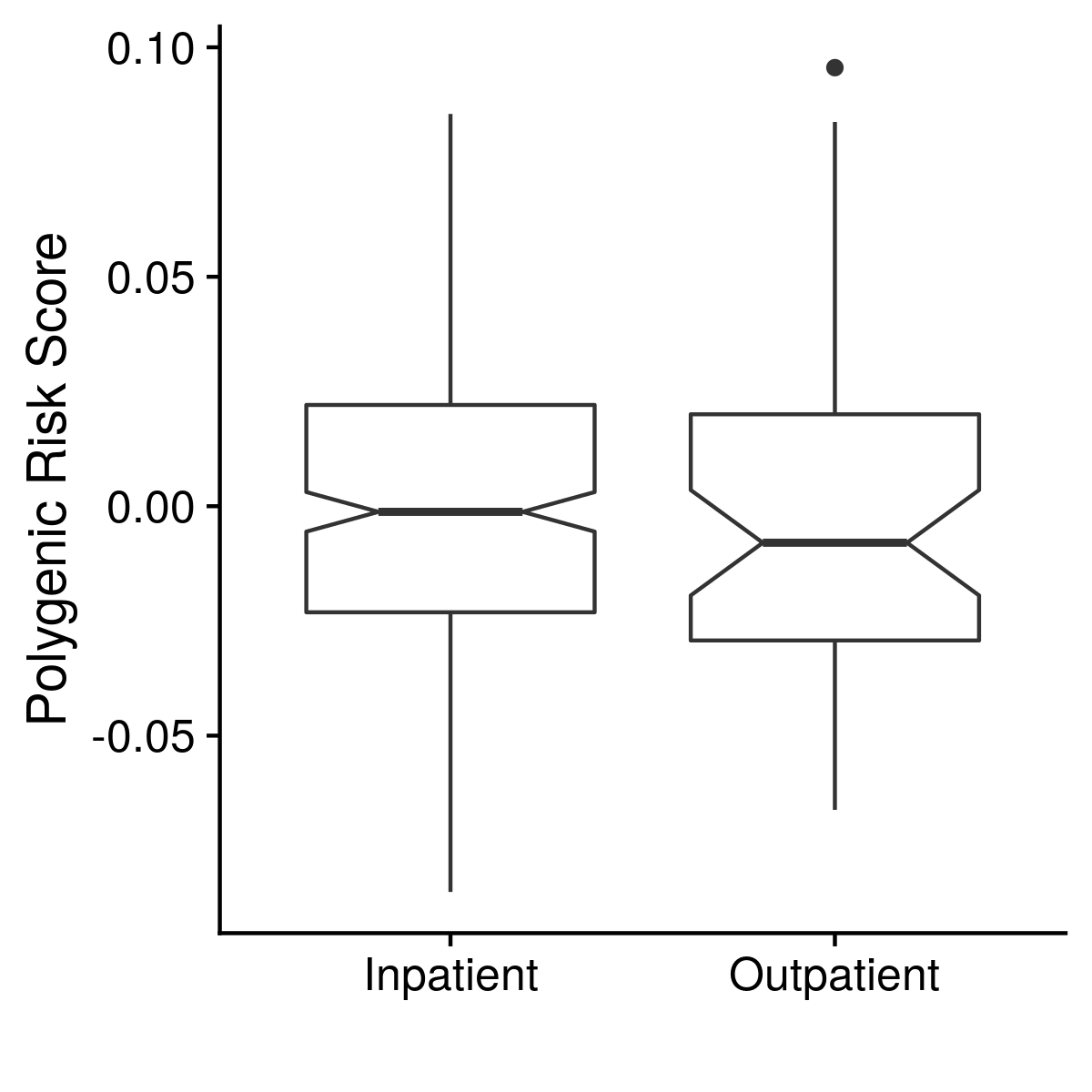


The entire population of participants who tested positive once was randomly split into training (47 controls and 269 cases) and testing (46 controls and 274 cases) datasets. The training data was used to perform a genome wide association study as previously described. The result summary statistics were clumped with a p-value threshold of 0.01 and linkage disequilibrium R^2^ threshold of 0.5. Polygenic risk scores generated for the testing dataset and the inpatient status were compared. The difference between the polygenic risk scores of the outpatient and inpatient groups was determined to be insignificant (P = 0.99, T-test).

**References**

1. Groot, H. E. *et al.* Genetically Determined ABO Blood Group and its Associations With Health and Disease. *Arteriosclerosis, Thrombosis, and Vascular Biology* **40**, 830–838 (2020).
